# Comparative Chemical Profiling and Citronellol Enantiomers Distribution of Industrial-Type Rose Oils Produced in China

**DOI:** 10.3390/molecules28031281

**Published:** 2023-01-28

**Authors:** Ana Dobreva, Daniela Nedeltcheva-Antonova

**Affiliations:** 1 Institute of Roses and Aromatic Plants, Agricultural Academy, 6100 Kazanlak, Bulgaria; 2 Institute of Organic Chemistry with Centre of Phytochemistry, Bulgarian Academy of Sciences, 1113 Sofia, Bulgaria

**Keywords:** *R. damascena* Mill., *R. rugosa*, Kushui rose, oil-bearing roses, gas chromatography-mass spectrometry, chemical profiling, citronellol enantiomers separation

## Abstract

The chemical composition and aroma profile of industrial essential oils (EOs) from species of rose grown in China, including the native Kushui rose (*R. sertata* × *R. rugosa*) and *R. rugosa* Thunb. cv. Plena, and the recently introduced Damask rose (*R. damascena* Mill.), were studied in comparison by means of GC/MS and GC-FID. More than 150 individual compounds were detected in Chinese rose samples, of which 112 were identified and their quantitative content determined, representing 88.7%, 96.7% and 97.9% of the total EO content, respectively. It was found that the main constituents of the Chinese rose EOs were representatives of terpenoid compounds (mono- and sesquiterpenoids, predominantly) and aliphatic hydrocarbons. Comparative chemical profiling revealed different chemical composition and aroma profiles: while the *R. damascena* oil showed a balance between the eleoptene and stearoptene fractions of the oil (the average ratio between the main terpene alcohols and paraffins was 2.65), in the Kushui and *R. rugosa* oils, the odorous liquid phase strongly dominated over the stearopten, with a ratio of 16.91 and 41.43, respectively. The most abundant terpene was citronellol, ranging from 36.69% in *R. damascena* to 48.32% in *R. rugosa* oil. In addition, the citronellol enantiomers distribution, which is an important marker for rose oil authenticity, was studied for the first time in *R. rugosa* oil.

## 1. Introduction

The genus *Rosa* L. (Rosaceae) comprises 200 species with more than 18,000 cultivars naturally spread throughout the temperate and subtropical regions of the Northern Hemisphere and 95 of them have been found in China [1]. The plant is highly valued for its decorative features, but also has high economic importance due to the essential oil in its petals. Only a few rose species have found industrial-scale applications for their fragrance and flavoring properties: *Rosa damascena* Mill. var. *trigintipetala* Dieck., *Rosa gallica* L., *Rosa centifolia* L. and *Rosa alba* L. [2], widely used for the production of rose oil, rose water, absolute and concrete. The most important rose species from a commercial point of view, cultivated for rose oil production, is *R. damascena* Miller. It is considered superior in terms of essential oil quality and is produced in Bulgaria, Turkey, Iran, Saudi Arabia and India. Rose oil is an important raw material in the flavor and fragrance industry, but it is also widely used in cosmetology, clinical aromatherapy, and medicine due to its benefits to human health. Rose species have a wide spectrum of bio-pharmacological activities such as antidepressant, hypoglycemic, cardiovascular, anti-inflammatory, analgesic, antioxidant and antimicrobial effects [3,4,5,6,7,8].

Roses have been grown in East Asia for thousands of years. The most ancient local species are *Rosa chinensis, Rosa multiflora* and *Rosa rugosa* Thunb. [1,9,10]. *R. rugosa* is an oil-bearing plant with a small number of flower petals, but as a result of the long-term work of Chinese breeders, cultivars with multi-petal blossoms and a high content of essential oil were developed. The most cultivated species is *R. rugosa* Thunb. cv. plena [1,11], widespread in the eastern provinces, with a planting area more than 4000 hectares. It is known as the Pingyin rose (named after Pingyin county in Jinan, East China’s Shandong province) and has been used as material for industrial rose oil since the beginning of the XXth century. The flowers are large, with thick petals that are violet-pink in color. Its odor is strong and sweet. 

Second in importance, but even more famous, is the so-called Kushui rose, which is grown in the central provinces of China (Gansu). It is authorized as a natural hybrid of *R. sertata* × *R. rugosa*, but without any direct genetic evidence [1,12]. Its essential oil is highly valued and its annual production averages 600–700 kg [13]. The limited variation in its major components are defined by national and international standards [10,14]. It is worth mentioning that Kushui rose oil is the second rose oil, after *R. damascena* oil, with an ISO standard for quality and authenticity [14]. 

With the country’s century-old traditions in rose cultivation, in recent decades, China has started to develop its own rose oil production based on *R. damascena* [2,10] by introducing the species into provinces with suitable climatic conditions [15,16]. Due to the large-scale development of production technology, the country has earned its place in the world market as one of the producers of *R. damascena* oil [17].

Although its botanical origin (i.e., rose species genotype) is the main factor determining the quantitative and qualitative characteristics of rose essential oil and its aromatic products, there are many other factors affecting the chemical compositions and quality of the final product: geographical origin (climatic and soil conditions), flower processing technology (storage conditions and production methods) [18], local traditions and even the analytical techniques used are all of significant importance [2,19,20,21,22,23]. 

Feng et al. studied the chemical composition of *R. rugosa* Thunb. flowers in different developmental stages [11]. Fifty-three compounds were identified in the flower-bud stage, sixty-five at the early opening stage, sixty-two during half opening, sixty-five at full opening, and fifty-eight at the end of the full opening stage. The main aroma constituents were already formed during the early opening stage, with the majority of them reaching peak concentration between the half and full opening stages, when they should be picked [11,24]. Wua et al. [25] reported that salt intervention during flower processing increases the essential oil yield by 56.52% and the citronellol content by 31.90%, while the geraniol content decreases by 16.34%.

An analysis of the aroma constituents present in the Chinese Kushui-type rose essential oil by GC-FID and GC-MS was performed using three types of capillary columns with different polarities. The main constituents, as determined by GC-FID, were β-citronellol (41.6~46.7%), geraniol (9.7~11.0%), and nerol (3.4~4.5%). In addition, a comparative analysis was conducted using Bulgarian rose (*R. damascena* Miller) oil and perfume; Bulgarian rose oil showed a richer profile of characteristic aroma constituents than the Chinese Kushui-type essential oil [26]. Double molecular distillation was used to separate different fractions from Kushui rose oil and to evaluate the antioxidant and antimicrobial activity. Citronellol and nerol were considered to be the characteristic aroma components with differences of 3.57–34.9% and 0.29–5.99%, respectively, between the fractions [18]. 

A comparative analysis of the headspace volatiles of Chinese *R. rugosa* was performed, in which volatile fractions of 23 rose germplasms were isolated and up to 33 volatile compounds were identified, including 2-phenylethanol, β-citronellol, ethanol, and n-hexane [27]. The chemical composition of *R. rugosa* Thunb. var. plena Regal oil was studied by Ueyama et al. [23], revealing high citronellol content (60%). 

Solid-phase micro extraction (SPME)-headspace analysis with olfactometric evaluation revealed the compounds responsible for the characteristic odor of the Chineese *R. damascena* oil and the changes in its composition after application on human skin [19]. 

The key aroma compounds of industrial oils from *R. damascena*, *R. centifolia*, *R. alba*, *R. rugosa* cv. ‘Plena’ and *Rosa xanthina* Lindl were studied using gas chromatography–olfactometry (GC–O), gas chromatography–mass spectrometry (GC–MS) and quantitative descriptive analysis (QDA) [28]. Chinese rose concrete was steam-distilled to essential oil and its composition was compared with the rose absolutes from Bulgaria, France and Japanese *R. rugosa* Thunb [20].

Although there are some data on the chemical composition and olfactometric characteristics of the essential oils from various rose species grown in China, comprehensive chemical profiling of industrial-type essential oils from the main oil-bearing roses has not been performed up to now. It is worth underlining that no data on the enantiomer distribution of the important constituents of rose oil, such as citronellol, were found in the literature.

Therefore, the main aim of the current study was to perform a comparative chemical profiling of essential oils from the main oil-bearing rose species with industrial importance produced in China, revealing new data on the chemical composition and chirality of some important aroma profile components. 

## 2. Results and Discussion

**Figure 1:**
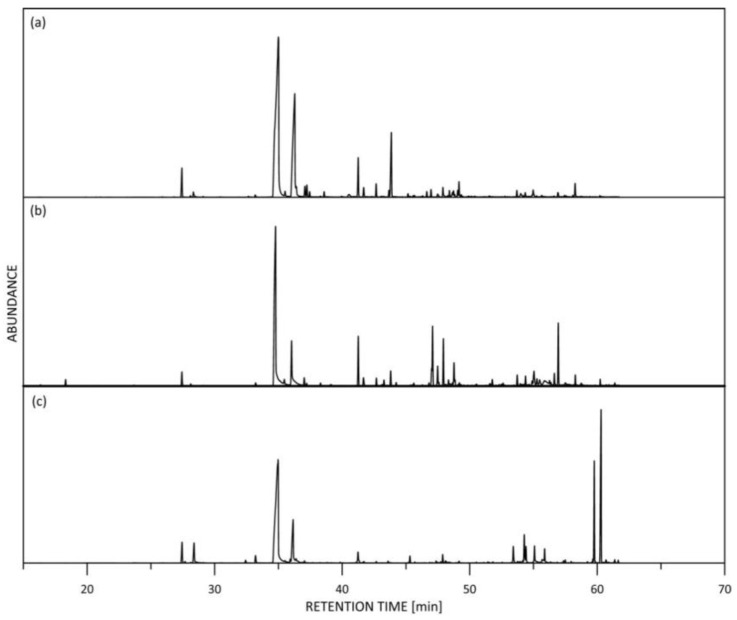
GC/MS (TIC) chromatograms of the Chinese RO samples: (**a**) *R. rugosa*; (**b**) Kushui rose; (**c**) *R. damascena*.

**Figure 2:**
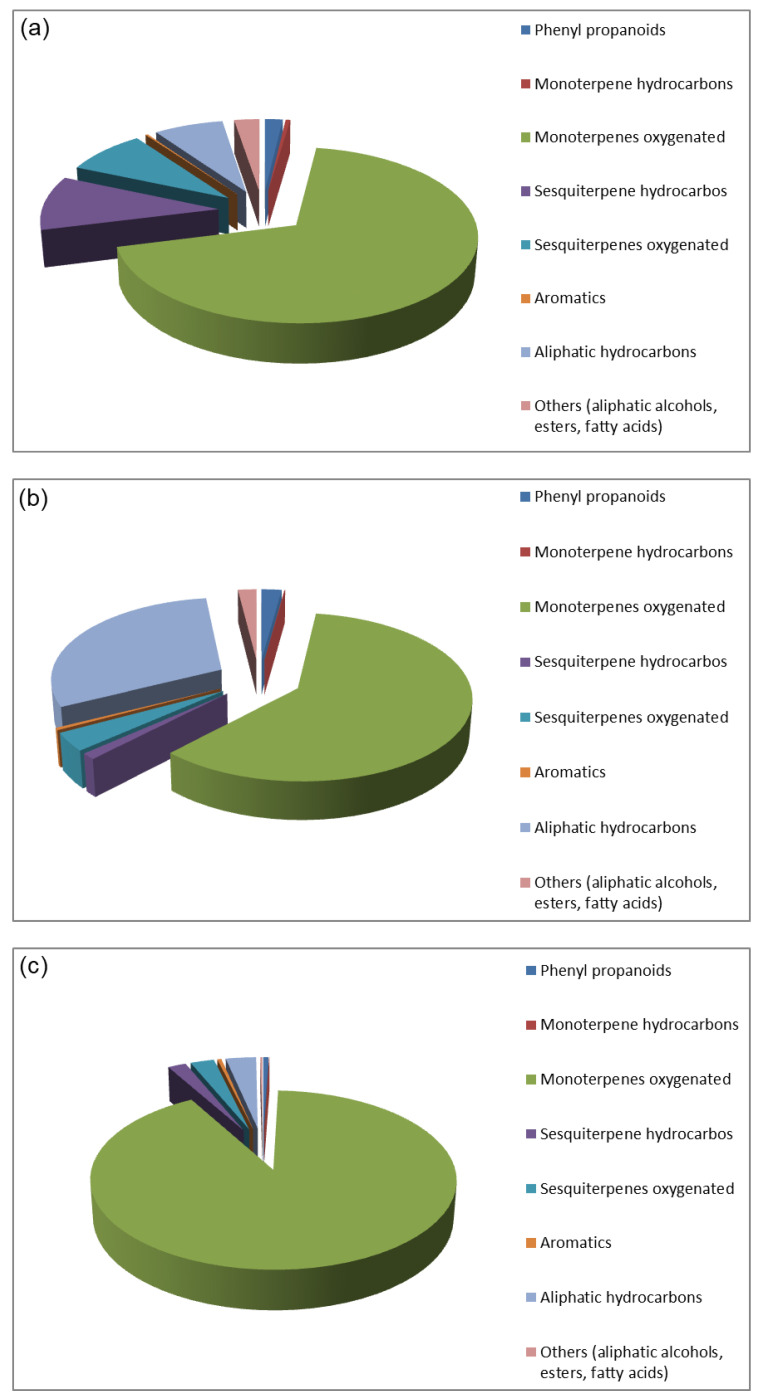
Main chemical classes distribution of the Chinese rose oil samples: (**a**) Kushui rose, (**b**) *R. damascena*, (**c**) *R. rugosa*.

Rose oil is a very complex mixture containing compounds with high structural diversity. More than 150 individual compounds were detected in Chinese rose essential oil samples, 112 of which were identified by GC/MS, and their quantitative content was determined by GC-FID, representing 96.74–99.40% of the total essential oil content in D1-D4 samples, 88.74% in KS- and 96.71% in R-sample. Quantitative data for the components of the Chinese rose oil samples, as determined by GC-FID, with concentrations higher than 0.01%, are summarized in Table 1. As seen from the data in Table 1, the main constituents of the Chinese rose oil samples are representatives of the terpenoids compounds (mono- and sesquiterpenoids, predominantly) and aliphatic hydrocarbons. The representative GC/MS chromatograms in the total ion current (TIC) mode of the samples are shown in Figure 1. The clearly distinguished chromatographic fingerprint of the Chinese rose oil samples can be seen. The distribution in the main chemical classes in the samples is presented in Figure 2. 

### 2.1. Terpenes

Terpenes and terpenoids are the main biosynthetic building blocks and important mediators of ecological interactions in plants. Of all the terpenoids, the mono- and sesquiterpenes are the main constituents of the EOs and are most frequently studied due to their abundance.

#### 2.1.1. Monoterpenes

*Alcohols.* As seen from Table 1, the most abundant class of aroma compounds found in the Chinese rose oils are monoterpene alcohols. β-citronellol, determining the basic rosaceous character of the rose oil, was found in a concentration range of 36.69–39.09% in Damask rose, 37.61% in Kushui rose, and the highest content in *R. rugosa* oil at 48.32%. These results are in agreement with the literature data, defining *R. rugosa* oil produced in China as a high citronellol type [6,26,29]. Similar results were also reported for Kushui rose oil at 39.5% [25], while the citronellol content in industrial *R. damascena* EO was found in a lower concentration in the range 5.04–18.87%, which can be attributed to the ecological conditions [21] or the local production technology features [19].

Geraniol was observed in the range 7.55–8.78% in D1-D4 and KS samples, and in a much higher concentration at 19.88% in R samples, which is in agreement with other data [15,21].

Various pharmacological activities are reported for these two main terpene alcohols, such as antispasmodic, anti-inflammatory, antibacterial and antifungal effect (citronellol), and insecticidal, repellent, acaricidal, antibacterial, and antifungal activity (geraniol) [5].

The concentration of nerol did not show significant differences between *R. damascena* and *R. rugosa* samples (5.94–6.77%), while in Kushui rose oil it was only 2.97%.

These three terpene alcohols are responsible for the specific odor of the rose oil: citronellol with a pleasant rose-like odor, geraniol with a flowery rose-like odor (different from that of citronellol), and nerol with a pleasant rose-like odor (different from that of geraniol). The observed high concentration of these important terpene alcohols in Chinese rose EOs reveals their olfactory and health-beneficial potential.

Other monoterpene alcohols found in the Chinese rose oils were linalool at a concentration between 1.49% (KS) and 2.13% (D1), and terpinen-4-ol and α-terpineol, which have been observed in a relatively constant concentration in D1-D4 samples (0.22% and 0.62%, respectively). 4-Terpineol was observed in tr. amount (<0.1%) in KS and R samples.

*Oxides.* Rose oxide has four isomers, of which the (-)-cis isomer is the most desirable for its fragrance properties. Cis- rose oxide was found in Chinese rose oils in low amounts, with the highest concentration in KS (0.19%) and R (0.11%), and its content complies with the requirements of the ISO 25157:2013 standard (<0.5%) [14]. Trans-rose oxide and nerol oxide was found in trace amounts (<0.1%).

*Aldehides.* Among the acyclic monoterpene aldehydes, citronellal and citrals are the key aroma compounds in many Eos, with an odor reminiscent of lemon. In Chinese rose oils, citronellal was found in the highest concentration in the KS sample (1.42%).

*Monoterpene hydrocarbons* are present in very low, trace amounts in all samples, with the highest quantity in Kushui rose oil (0.42%).

#### 2.1.2. Sesquiterpenes

*Sesquiterpene hydrocarbons* were most abundant in the KS sample (8.98%), with the main representatives γ-cadinene (3.35%), β-cubebene (2.70%), α-amorphene (1.14%), γ-muurolene (0.81%), etc., while the total amount of the sesquiterpene hydrocarbons in other samples were in the range 1.19–1.69%. Daucene (*trans*-dauca-4,8-diene) and isodaucene (dauca-8,11-diene) are sesquiterpene hydrocarbons that have not been reported in *R. damascena* oils. In this study, these compounds were found in amounts of 0.34% and 1.26% (KS) and in trace amounts of (<0.01%) and 0.24% (R), respectively, and could be used as marker compounds for the botanical origin of Kushui and *R. rugosa* oil. This observation is in agreement with the latest studies on Pinguin rose oil [6].

*Oxygenated sesquiterpenes*. The sesquiterpene alcohol trans-β-farnesol was found in the highest amount in KS (1.76%) in the range 0.51–0.60% (D1-D4), and in trace amounts in R-samples. -A- and β-eudesmols are other sesquiterpene alcohols typically observed in *R. damascena* oil. In this study, they were found in D1-D4 samples at a concentration of 0.93% and 1.44%, respectively, and only in trace amounts in KS and R samples.

The tricyclic sesquiterpene ketone *germazone* is another interesting compound previously reported in *Geranium macrorrhizum* L. [30] that had not been found until now in rose oils. It was observed in amounts of 0.53% in Kushui oil, 0.74% in *R. rugosa* oil and in trace amounts (0.1%) in D1-D4 samples.

### 2.2. Aliphatic hydrocarbons (Stearopten)

*Aliphatic hydrocarbons (alkanes and alkenes*), although odorless, play a significant role in rose oils as compounds responsible for odor stability. The content of heptadecane and nonadecene/nonadecane is considered to be of particular importance for the quality of *R. damascena* oil. In the Chinese rose oil samples, heptadecane was found in the range 1.44 (KS) and 1.60% (D1-D4 samples), while in R samples, it was detected only in trace amounts (<0.01%). It is interesting to mention that nonadecane/nonadecene was not observed in KS ad R samples. In general, the total amount of aliphatic hydrocarbons was found to be relatively low in Kushui and *R. rugosa* oils at 6.23% and 2.80%, respectively, in contrast with *R. damascena* oils (28.71–31.49%).

**Figure 3:**
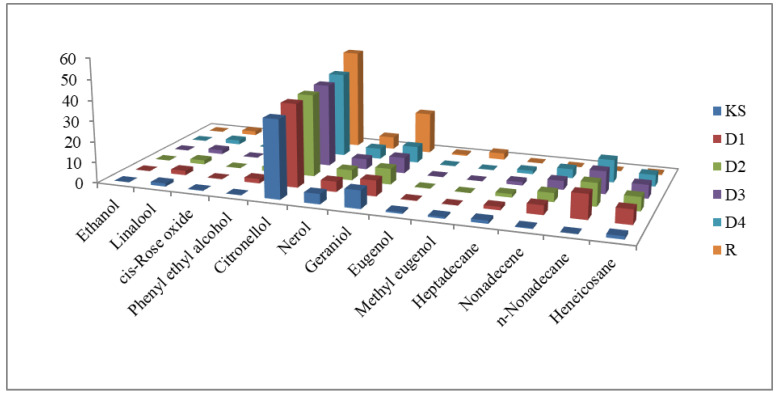
The most important Chinese rose oil components, monitored by the ISO, as determined by GC-FID.

The most important components in rose oil, responsible for rose oil quality and authenticity, are regulated by the International Standard Organization (ISO). Table 2 and Figure 3 present quantitative data determined by GC-FID, together with the ISO 9842:2003 and ISO 25157:2013 specifications. The qualitative and quantitative analyses of the aroma constituents reveal that *R. damascena* EOs are, in general, not consistent with the International standard ISO 9842:2003 [31]: they do not fit into the parameters for the Bulgarian rose oil, but show similar characteristics to oils originating from Turkey and Morocco. The chemical profiles of Kushui and *R. rugosa* rose oils differ most significantly.

It is interesting to mention that the chemical composition of the KS and R samples do not fully comply with the ISO 25157:2013 standard for Kushui rose oil produced in China [14]. It is noteworthy that the document limits some minor components whose values vary in a narrow interval of concentration; for a natural product such as rose oil, this is difficult to achieve. Therefore, it can be assumed, in general, that the composition of the KS sample fits into the standard (with the exception of geranyl acetate). The other authors also note similar deviations, both for macro and minor constituents [18,25,26]. Regarding the chemical compositions of the R sample, our results are comparable with other investigations of the same genotype [6,23,29]. It is noteworthy that in all studies, the methyl eugenol content is above 3%, which is in agreement with our findings (3.11%) and can be attributed to the genotype specificity.

The relationship between the content of terpene alcohols and aliphatic hydrocarbons can also be easily traced through the standard. This ratio is an important indicator for the perfumery quality of rose oil. The ingredients of the odoriferous liquid part (eleoptene) determine the overall aroma character, while the odorless paraffins are responsible for its durability. In our study, the *R. damascena* oils revealed a total amount of main terpene alcohols citronellol + nerol + geraniol, on average, of 52.35%, and 19.41% for the main aliphatic hydrocarbons C_17_ + C_19_ + C_21,_ with the ratio of 2.65. Compared with the ISO 9842:2003 data, this value is close to Bulgarian rose oil (2.91), but differs from the Turkish and Moroccan oils (with ratio of 4.34 and 3.42, respectively).

In contrast, a low paraffin content was observed in the Kushui and *R. rugosa* oil samples, demonstrating a terpene alcohols:paraffins ratio of 16.91 and 41.43, respectively (27.69 in the ISO 25157:2013 standard). This fact could be explained by the peculiarities of its blossoms and the thinner wax layers.

### 2.3. Enantiomers Distribution

It is worth noting that plants produce metabolites in many instances, such as chiral molecules, and enantiomers can differ from one species to another within the same genus. Although presenting the same physicochemical properties, except for their optical activity, enantiomers can exhibit divergent biological activities [32], including different aroma properties. Therefore, it is of crucial importance for essential oil quality and authenticity that the exact enantiomer distribution of important odor-bearing compounds are investigated. Citronellol exists in nature as two enantiomers, namely R-(+) citronellol and S-(–) citronellol. The (–) isomer is naturally presented in *R. damascena* and the citronellol enantiomer ratio in *R. damascena* oil is monitored by the European Pharmacopoeia [33], requiring excess S-(–) citronellol of >99% and R-(+) citronellol should be presented in amounts <1%.

According to our observations, D1-D4 and KS samples comply with these requirements.

An interesting hypothesis was drawn by Wu et al. [25], who investigated the influence of salt intervention on the composition, aroma, and antioxidant properties of Kushui rose oil: the authors reported increasing the R-(+) citronellol content when rose petals were processed by salinization, explaining this finding by the higher boiling point of the R-(+) citronellol.

Within the frame of this study, the citronellol enantiomer distribution was studied for the first time in *R. rugosa* oil, revealing 82.9% S-(–) citronellol and 17.1% R-(+) citronellol content in R samples. Although this observation is very interesting and could be used for botanical origin assessment, more *R. rugosa* oil samples should be analyzed to confirm these preliminary results.

**Figure 4:**
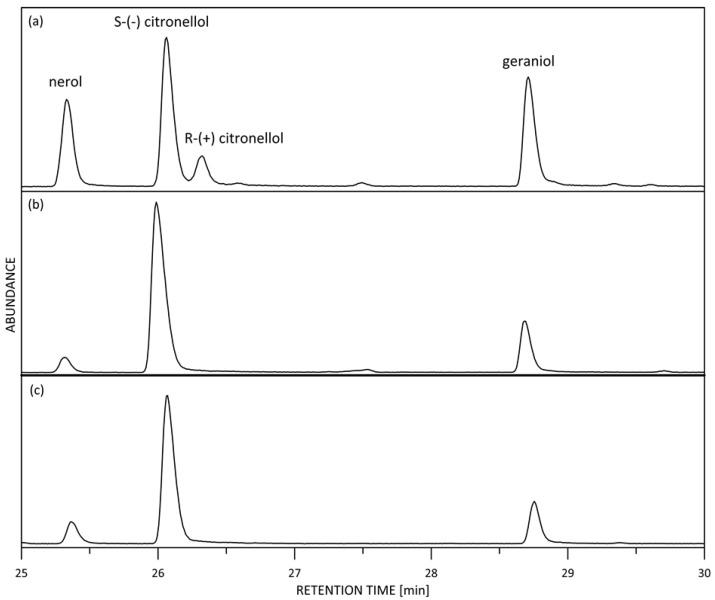
GC/MS (TIC) chromatograms on a chiral stationary phase Cyclodex-B: (**a**) *R. rugosa*; (**b**) Kushui rose; (**c**) *R. damascena*.

The GC/MS (TIC) chiral chromatograms of the Chinese rose oil samples are presented in Figure 4.

## 3. Materials and Methods

### 3.1. Samples

Six samples of industrial-type rose EOs were studied: four rose oil samples of *R. damascena* Mill. from Uyghur (Xiujiang), provided by Shenzhen BioTech Ltd., a sample of essential oil from the Kushui rose (*R. sertata* × *R. rugosa*), provided by the Gansu Agricultural University, Gansu Province, and a sample of essential oil from the Pingyin rose (*R. rugosa* cv. Plena), provided by Jinan Huinong Rose Essential Oil Co., Ltd. According to the suppliers, the age of the plantations is 5–10 years, cultivated according to the established technology. The Kushui and *R. rugosa* rose oil samples were derived in 2017 and *R. damascene* in 2019. In this manuscript, the *R. damascena* samples are marked as D1-D4 and the Kushui and the *R. rugosa* samples as KS and R, respectively.

For the citronellol enantiomers study, the following reference materials were used: (R)-(+)-*β*-citronellol, 97% and citronellol, mixture of isomers, natural, ≥95%, FG (Sigma-Aldrich, Saint Louis, MO, USA).

For the Retention indices calculation, a standard mixture of aliphatic hydrocarbons from C8 to C32, >98%, dissolved in GC grade n-hexane (Sigma-Aldrich, Saint Louis, MO, USA), was used.

### 3.2. Analytical Methods

Gas chromatography-mass spectrometry (GC/MS) and gas chromatography with flame ionization detection (GC-FID) were used for the chemical profiling. In addition, GC/MS with chiral stationary phases was applied and the distribution of citronellol enantiomers was evaluated.

The GC/MS analysis was performed on an Agilent 7820A GC System gas chromatograph coupled with a 5977B Mass Selective detector and flame-ionization detector (Agilent Technologies, Palo Alto, CA, USA). The ultra-inert non-polar fused silica capillary column DB-5ms UI (J&W Scientific, Folsom, CA, USA) with 30 m column length, 0.25 mm i.d. and 0.25 mm film thickness was used. 

The oven temperature was programmed from 60 °C (2.5 min held) to 100 °C at a rate of 5 °C/min, from 100 to 225 °C at a rate of 2.5 °C/min, and from 225 to 275 °C at a rate of 5 °C, 10 min held at the final temperature. Helium (99.999%) was used as a carrier gas at a constant flow rate of 0.8 mL/min. The split ratio was 1:200, the inlet temperature was set to 260 °C and the transfer line temperature was 280 °C. A mass selective detector was operated in electron impact ionization (EI) mode at 70 eV electron energy, the ion source temperature was set to 230 °C, and the quadrupole temperature was 150 °C. The mass scan range was 30–600 *m*/*z*.

The GC-FID analysis was performed on the same instrument under the same temperature gradient as described above. The system was equipped with a post-column split of the flow, allowing the simultaneous analysis of both detectors. Instrument control and data collection were carried out using Mass Hunter Workstation Software (Revision B.06.07, Agilent Technologies).

The identification of the compounds was performed using commercial mass spectral libraries (NIST 14, Wiley 7th Mass Spectra Register) and retention times (linear retention indices, LRI). In cases where there was a lack of corresponding reference data, the structures were proposed based on their general fragmentation pattern or using reference literature mass spectra. The quantification of the main compounds was carried out by the internal normalization method, with a response factor equal to unity for all of the sample constituents.

Retention indices were calculated using a mixture of homologues aliphatic hydrocarbons (C8-C32) analyzed under identical conditions, applying the following equation [34]:

RI (x) = 100 n + 100 [(t_R_^x^ − t_R_^n^)/(t_R_^n+1^ − t_R_^n^)],

where: t_R_^x^—retention time of the component x; t_R_^n, n+1^—retention times of the n-alkanes, eluting before and after the component × (bracketing x); n, n + 1—carbon atom number of the n-alkanes, eluting before and after the component x.

For the enantiomers distribution, chiral stationary phase Cyclodex-B, with 30 m column length, 0.25 mm i.d., 0.25 mm (J&W Scientific, Folsom, CA, USA) was used under the following temperature gradient: from 60 °C (2.5 min held) to 120 °C at a rate of 2.5 °C/min (4.5 min held) and from 100 to 225 °C at a rate of 10 °C/min 10 min isotherm at the final temperature.

## 4. Conclusions

A comparative study of Chinese rose oils revealed their distinctive chemical composition and aroma profile, both for introduced and native rose genotypes. The main constituents of the Chinese rose oil samples are representatives of terpenoids compounds (mono- and sesquiterpenoids, predominantly) and aliphatic hydrocarbons. Higher amounts of characteristic aroma constituents in the EOs from Damask rose were observed in comparison with the Kushui rose and *R. rugosa* oils. In addition, the chemical profile of Chinese *R. damascena* oil samples differs significantly from the Bulgarian one, but is close to the Turkish and Moroccan rose oil types (ISO 9842:2003).

## Figures and Tables

**Table 1:** Chemical composition of the rose oils samples, as determined by GC/MS/FID on a DB-5ms column.

No	Compounds	Formula	Mw	RI_ref_^1^ (DB5)	RI_exp_ (DB5)	Rel.%, as Determined by GC-FID					
						D1	D2	D3	D4	KS	R
1.	Ethanol	C_2_H_6_O	46	459	n.d.	0.03	0.03	0.02	0.04	0.09	0.01
2.	β-Pinene	C_10_H_16_	136	974	981	n.d. ^2^	n.d.	n.d.	n.d.	0.08	0.01
3.	β-Myrcene	C_10_H_16_	136	988	987	0.1	tr. ^3^	0.01	tr.	0.03	0.02
4.	Furane, 2-pentyl-	C_9_H_14_O	138	984	988	0.1	tr.	n.d.	n.d.	0.02	0.01
5.	p-Cymene	C_10_H_14_	134	1022	1027	0.1	tr.	tr.	n.d.	0.02	tr.
6.	Limonene	C_10_H_16_	136	1024	1032	0.03	0.04	tr.	tr.	0.05	tr.
7.	Benzyl alcohol	C_7_H_8_O	108	1026	1033	0.04	0.02	0.05	0.04	n.d.	n.d.
8.	α-Ocimene	C_10_H_16_	136	1032	1037	n.d.	n.d.	n.d.	n.d.	0.11	n.d.
9.	β-Ocimene	C_10_H_16_	136	1044	1045	n.d.	n.d.	n.d.	n.d.	0.07	0.01
10.	Linalool oxide	C_10_H_18_O_2_	213	1067	1073	0.04	0.04	0.03	0.03	0.03	0.06
11.	α-Terpinolene	C_10_H_16_	136	1086	1089	0.02	0.01	0.01	0.01	0.06	n.d.
12.	Linalool	C_10_H_18_O	154	1095	1099	2.13	1.98	2.02	2.06	1.49	1.98
13.	Nonanal	C_9_H_18_O	142	1100	1104	0.17	0.16	0.16	0.16	0.06	n.d.
14.	*cis*-Rose oxide	C_10_H_18_O	154	1106	1112	0.09	0.06	0.07	0.07	0.19	0.11
15.	Phenyl ethyl alcohol	C_8_H_10_O	122	1106	1116	2.13	2.07	2.09	2.13	0.05	0.47
16.	*trans*-Rose oxide	C_10_H_18_O	154	1122	1129	0.04	0.05	0.04	0.04	0.07	0.06
17.	Nerol oxide	C_10_H_16_O	152	1154	1153	0.05	0.03	0.04	0.03	0.07	0.04
18.	Camphor	C_10_H_16_O	152	1141	1155	0.02	0.02	0.02	0.02	tr.	0.01
19.	4-Terpineol	C_10_H_18_O	154	1174	1191	0.22	0.21	0.22	0.22	0.03	0.08
20.	α-Terpineol	C_10_H_18_O	154	1200	1200	0.63	0.61	0.62	0.63	0.36	0.16
21.	α-Ionene	C_13_H_18_	174	1255	1220	0.04	tr.	tr.	tr.	tr.	0.04
22.	p-Menth-1-en-9-al	C_10_H_16_O	152	1232	1224	0.07	0.03	0.03	n.d.	n.d.	0.02
23.	Citronellol	C_10_H_20_O	156	1223	1232	39.01	38.51	36.69	39.09	39.51	48.32
24.	Nerol ^4^	C_10_H_18_O	154	1227	n.d.	6.35	6.77	5.94	6.36	2.97	5.97
25.	Citronellal	C_10_H_18_O	154	1165	1241	0.26	0.49	0.34	0.46	1.40	0.42
26.	Neral	C_10_H_16_O	152	1235	1242	0.42	0.38	0.48	0.27	tr.	tr.
27.	Geraniol	C_10_H_18_O	154	1249	1249	7.55	7.59	7.64	7.88	8.66	19.88
28.	Linalyl acetate	C_12_H_20_O_2_	196	1254	1253	0.28	0.37	0.36	0.30	n.d.	n.d.
29.	Phenyl ethyl acetate	C_10_H_12_O	164	1258	1258	0.46	0.78	1.00	0.99	053	1.78
30.	Geranial	C_10_H_16_O	152	1264	1269	0.19	0.19	0.20	0.18	0.91	0.76
31.	Citronellyl formate	C_11_H_20_O_2_	184	1271	1273	0.04	0.02	0.03	0.03	0.21	0.55
32.	Neryl formate	C_11_H_18_O_2_	182	1280	1281	0.03	0.02	0.03	0.03	tr.	0.25
33.	*trans*-Anethole	C_10_H_12_O	148	1282	1291	0.03	0.02	0.02	0.02	tr.	0.06
34.	2-Undecanone	C_11_H_22_O	170	1293	1292	0.01	0.01	0.01	0.01	0.27	tr.
35.	Geranyl formate	C_11_H_18_O_2_	182	1298	1297	0.10	0.13	0.13	0.12	tr.	0.29
36.	Citronellyc acid	C_12_H_22_O_2_	198	1312	1302	0.14	tr.	tr.	0.14	tr.	tr.
37.	2-Methylnaphthalene	C_11_H_10_	142	1310	1316	tr.	0.02	0.02	0.02	0.42	n.d.
38.	Methyl geranate	C_10_H_16_O_2_	168	1322	1322	0.06	0.06	0.06	0.06	0.07	0.01
39.	Methylnaphthalene (isomer)	C_11_H_10_	142	1318	1324	tr.	0.01	0.01	tr.	0.03	0.11
40.	Citronellyl acetate + Geranic acid ^5^	C_12_H_22_O_2/_C_10_H_16_O_2_	198/168	1350	1350	1.32	1.00	1.24	1.29	3.03	1.76
41.	Eugenol + Neryl acetate	C_10_H_12_O_2/_C_12_H_20_O_2_	164/196	1356	1358	0.15	0.14	0.15	0.14	0.74	0.63
42.	Geranyl acetate	C_12_H_20_O_2_	196	1379	1378	0.11	0.11	0.11	0.11	0.58	0.66
43.	α-Copaene	C_15_H_24_	204	1374	1381	0.02	0.02	0.02	0.02	0.13	0.06
44.	Daucene	C_15_H_24_	204	1380	1390	0.01	0.01	0.01	0.01	0.34	tr.
45.	2-Dodecanone	C_12_H_24_O	184	1388	1394	n.d.	n.d.	n.d	0.01	0.07	tr.
46.	β-Bourbonene	C_15_H_24_	204	1388	1396	0.11	0.12	0.12	0.11	tr.	tr.
47.	β-Elemene	C_15_H_24_	204	1389	1398	tr.	tr.	tr.	tr.	tr.	0.28
48.	Methyl eugenol	C_11_H_14_O_2_	178	1403	1402	0.03	0.03	0.03	0.03	0.81	3.11
49.	α-Bisabolene	C_15_H_24_	204	1500	1422	tr.	tr.	tr.	0.01	0.04	0.02
50.	β-Gurjunene	C_15_H_24_	204	1431	1431	0.03	0.03	0.03	0.04	0.03	0.16
51.	*trans*-β-Caryophyllene	C_15_H_24_	204	1455	1435	0.41	0.39	0.39	0.38	0.07	0.05
52.	α-Bergamotene	C_15_H_24_	204	1432	1442	tr.	tr.	tr.	tr.	tr.	0.10
53.	Germacrene D	C_15_H_24_	204	1451	1444	0.06	0.07	0.06	0.06	n.d.	0.08
54.	Neryl acetone	C_13_H_22_O	194	1455	1450	0.02	0.02	0.02	0.02	0.03	0.02
55.	α-Humulene	C_15_H_22_	204	1464	1470	0.04	0.04	0.04	0.04	0.19	tr.
56.	γ-Muurolene	C_15_H_24_	204	1478	1475	0.04	0.04	0.04	0.04	0.81	0.30
57.	γ-Cadinene	C_15_H_24_	204	1513	1476	tr.	tr.	tr.	tr.	3.35	tr.
58.	Pentadecene	C_15_H_32_	212	n.d.	1483	0.12	0.12	0.13	0.12	n.d.	n.d.
59.	α-Amorphene	C_15_H_24_	204	1483	1486	0.05	0.05	0.05	0.05	1.14	0.15
60.	ar-Curcumene	C_15_H_22_	204	1479	1488	n.d.	n.d.	n.d.	n.d.	0.18	n.d.
61.	Phenyl ethyl 2-methyl butanoate	C_13_H_18_O_2_	206	1490	1491	0.07	0.07	0.07	0.06	0.04	0.02
62.	epi-Cubebol	C_15_H_24_*O*	220	1494	1495	0.46	0.43	0.44	0.43	2.70	0.49
63.	2-Tridecanone	C_13_H_26_O	198	1496	1496	0.02	0.02	0.02	tr.	tr.	0.06
64.	Pentadecane	C_15_H_32_	212	1500	1500	0.07	0.13	0.13	0.12	0.12	0.04
65.	Benzyl tiglate	C_12_H_14_O_2_	190	1497	1502	0.05	0.07	0.07	0.07	0.04	0.06
66.	α-Farnesene	C_15_H_24_	204	1505	1506	0.05	0.05	0.06	0.05	0.61	0.06
67.	γ-Muurolene	C_15_H_24_	204	1478	1509	0.06	0.06	0.06	0.05	0.35	0.27
68.	γ-Bisabolene	C_15_H_24_	204	1515	1515	0.02	0.02	0.02	0.02	0.26	tr.
69.	iso-Germacrene D	C_16_H_26_	218	n.d.	1516	n.d.	n.d.	n.d.	n.d.	n.d.	0.29
70.	Dauca-8,11-diene (Isodaucene)	C_15_H_24_	204	1500	1519	n.d.	n.d.	n.d.	n.d.	1.26	0.24
71.	α-Agarofuran	C_15_H_24_O	220	1548	1522	0.03	0.02	0.02	0.02	0.28	0.10
72.	α-Muurolene	C_15_H_24_	204	1500	1526	0.08	0.08	0.03	0.03	0.06	0.30
73.	δ-Cadinene	C_15_H_24_	204	1522	1529	0.01	0.09	0.09	0.08	0.15	0.52
74.	Nerolidol	C_15_H_26_O	222	1561	1565	0.06	0.07	0.07	0.07	0.22	0.04
75.	Hexadecene	C_16_H_32_	224	n.d.	1581	0.04	0.04	0.04	0.04	0.02	0.02
76.	Phenyl ethyl tiglate	C_13_H_16_O_2_	204	1584	1590	0.05	0.05	0.05	0.03	0.05	
77.	Ethyl laurate	C_14_H_28_O_2_	228	1594	1592	0.05	0.05	0.05	0.03	0.22	0.12
78.	Aromadendrene epoxide	C_15_H_24_O	220	1639	1599	0.08	0.08	0.08	0.08	0.44	0.05
79.	α-Cubenol	C_15_H_26_O	222	1645	1644	0.06	0.05	0.05	0.05	tr.	0.05
80.	α-Eugesmol	C_15_H_26_O	222	1649	1650	0.93	0.92	0.93	0.94	0.06	tr.
81.	Caryophylla-4(12),8(13)-dien-5α-ol	C_15_H_24_O	220	1639	1659	0.14	0.15	0.16	0.14	0.60	0.31
82.	α-Copaene-11-ol	C_15_H_24_O	220	n.d.	1666	0.07	0.06	0.08	0.07	0.19	0.07
83.	τ-Muurolol	C_15_H_26_O	222	1640	1672	0.13	0.15	0.14	0.13	0.14	0.10
84.	Bisabolol oxide	C_15_H_26_O_2_	238	1656	1671	n.d.	n.d.	n.d.	n.d.	n.d.	0.22
85.	α-Eudesmol	C_15_H_26_O	222	1652	1676	1.44	1.43	1.42	1.46	0.10	tr.
86.	Ledene oxide + Heptadecene	C_15_H_24_O/C_17_H_34_	220/238	n.d.	1680	0.81	0.84	0.83	0.81	0.61	tr.
87.	α-Bisabolol	C_15_H_26_O	222	1701	1698	n.d.	n.d.	n.d.	n.d.	n.d.	0.48
88.	2-Pentadecanone + Heptadecane	C_15_H_30_O/C_17_H_36_	226/240	1700	1700	1.59	1.67	1.66	1.66	1.44	tr.
89.	Nootkatol	C_15_H_22_O	218	1714	1707	n.d.	n.d.	n.d.	n.d.	n.d.	0.08
90.	*trans*-β-Farnesol	C_15_H_26_O	222	1714	1722	0.51	0.57	0.60	0.60	1.76	0.05
91.	Heptadecadiene	C_17_H_32_	236	n.d.	1729	0.98	0.93	0.92	0.93	n.d.	n.d.
92.	Farnesal	C_15_H_24_O	220	1715	1745	0.07	0.08	0.08	0.07	0.36	tr.
93.	Ylangenal	C_15_H_22_O	218	1764	1765	n.d.	n.d.	n.d.	n.d.	2.96	tr.
94.	Octadecene	C_18_H_36_	252	n.d.	1779	0.11	0.11	0.11	0.11	0.06	0.04
95.	Cinnamaldehyde, 3,4-dimethoxy-	C_11_H_12_O_3_	192	1790	1782	n.d.	n.d.	n.d.	n.d.	n.d.	0.13
96.	Benzyl benzoate	C_14_H_12_O_2_	212	1759	1772	0.13	0.12	0.13	0.13	0.15	0.11
97.	Ethyl myristate	C_16_H_32_O	256	1795	1792	0.02	0.02	0.02	0.02	0.11	n.d.
98.	Octadecane	C_18_H_38_	254	1800	1800	0.09	0.10	0.09	0.09	n.d.	tr.
99.	Germazone	C_15_H_22_O	218	1746	1814	0.02	0.01	0.01	0.01	0.53	0.74
100.	Phenyl ethyl octanoate	C_16_H_24_O_2_	248	1847	1856	0.13	0.12	0.13	0.14	n.d.	n.d.
101.	Nonadecene	C_19_H_38_	266	n.d.	1879	4.51	4.28	4.27	4.38	0.34	0.03
102.	n-Nonadecane	C_19_H_40_	268	1900	1900	11.82	11.08	10.94	10.87	tr.	tr.
103.	Eicosene	C_20_H_42_	282	n.d.	1976	0.11	0.12	0.12	0.11	tr.	0.02
104.	Eicosane	C_20_H_42_	282	2000	2000	0.84	0.80	0.79	0.80	0.11	0.02
105.	Geranic acid, 2-phenyl ethyl ester	C_10_H_16_O_2_	168	2081	2056	0.10	0.02	0.11	0.09	n.d.	0.08
106.	Heneicosane	C_21_H_44_	296	2100	2100	7.05	6.71	6.56	5.63	1.49	0.49
107.	Docosane	C_22_H_46_	310	2200	2200	0.27	0.25	0.20	0.25	0.08	0.08
108.	Tricosene	C_23_H_46_	322	n.d.	2289	0.02	0.02	0.04	0.02	0.31	0.09
109.	Tricosane	C_23_H_48_	324	2300	2300	2.33	2.21	1.67	2.10	1.34	1.30
110.	Tetracosane	C_24_H_50_	338	2400	2400	0.12	0.11	0.14	0.11	0.07	0.09
111.	Pentacosane	C_25_H_52_	352	2500	2500	0.43	0.41	0.31	0.39	0.21	0.46
112.	Heptacosane	C_27_H_56_	380	2700	2700	0.18	0.18	0.13	0.17	0.03	0.12
	Total Identified compounds					99.40	96.74	96.81	98.78	88.74	96.71
	Aliphatic hydrocarbons					31.49	30.11	29.08	28.71	6.23	2.80
	Aliphatics (alcohols, esters, fatty acids)					1.89	1.96	1.94	1.93	2.26	0.19
	Monoterpene hydrocarbons					0.25	0.05	0.02	0.01	0.42	0.04
	Monoterpenes oxygenated					58.84	57.64	58.38	60.82	59.58	81.39
	Aromatic-compounds					0.44	0.42	0.47	0.46	0.22	0.38
	Sesquiterpenes hydrocarbons					1.23	1.20	1.20	1.19	8.98	1.69
	Sesquiterpenes oxygenated					3.51	3.57	3.62	3.62	7.36	2.19
	Phenylpropanoids					2.34	2.26	2.29	2.32	1.60	4.27

*Remarks*: ^1^—according to NIST Chemistry WebBook, SRD 69 (https://webbook.nist.gov (accessed on 5 January 2023)); ^2^ n.d.—not detected; ^3^ tr.—traces <0.01%; ^4^—as determined on DB-17HT column; ^5^—co-eluting components.

**Table 2:** The most important Chinese rose oil components, together with the ISO 9842:2003 and ISO 25157:2013 specifications.

No	Compounds	Rel.%, as Determined by GC-FID					ISO 9842:2003 ^1^	KS	ISO 25157:2013 ^2^	R
		D1	D2	D3	D4	StDev(D)				
1.	Ethanol	0.03	0.03	0.02	0.04	0.01	≤2.0	0.09	1.0–3.5	Tr ^3^.
2.	cis-Rose oxide	0.09	0.06	0.07	0.07	0.01	n.d. ^4^.	0.19	≤0.5	0.11
3.	Linalool	2.13	1.98	2.02	2.06	0.06	≤2.0	1.49	1.0–3.5	1.98
4.	Phenylethanol	2.13	2.07	2.09	2.13	0.03	≤3.5	0.05	≤0.3	0.47
5.	Citronellol	39.01	38.51	36.69	39.09	1.12	20.0–34.0	39.51	40.0–50.0	48.32
6.	Nerol	6.35	6.77	5.94	6.36	0.34	5.0–12.0	2.97	2.0–5.5	5.97
7.	Geraniol	7.55	7.59	7.64	7.88	0.15	15.0–22.0	8.78	6.0–18.0	19.88
8.	Geranyl acetate	0.11	0.11	0.11	0.11	0.00	-n.d.	0.58	2.5–4.5	0.66
9.	Eugenol	0.15	0.14	0.15	0.14	0.01	≤2.0	0.74	n.d.	0.63
10.	Methyl eugenol	0.03	0.03	0.03	0.03	0.00	≤3.0	0.81	0.8–2.0	3.11
11.	Heptadecane ^5^	1.59	1.67	1.66	1.66	0.04	1.0–2.5	1.44	n.d.	tr.
12.	Farnesol	0.51	0.57	0.60	0.60	0.04	-n.d.	1.76	2.0–3.5	tr.
13.	Nonadecene	4.51	4.28	4.27	4.38	0.11	-n.d.	0.34	n.d.	tr.
14.	Nonadecane	11.82	11.08	10.94	10.87	0.44	8.0–15.0	n.d.	n.d.	tr.
15.	Eicosane	0.84	0.80	0.79	0.80	0.02	n.d.	0.12	n.d.	tr.
16.	Heneicosane	7.05	6.71	6.56	5.63	0.61	3.0–5.5	1.49	0.6–2.0	0.49
17.	Tricosane	2.33	2.21	1.67	2.10	0.29	n.d.	1.54	0.6–2.0	1.30
18.	Pentacosane	0.33	0.46	0.39	0.38	0.05	n.d.	0.21	n.d.	0.46
19.	Heptacosane	0.18	0.18	0.13	0.17	0.02	n.d.	<0.05	n.d.	0.12

*Remarks*: ^1^—International standard for *R. damascena*, produced in Bulgaria [31]; ^2^—International Standard for Kushui rose, produced in China [14]; ^3^ tr.—traces <0.01%; ^4^ n.d.—no data in the standard; ^5^—co-eluting with 2-Pentadecanone.

## Data Availability

The data presented in this study are available in this article.
